# Efficacy of Regorafenib in Hepatocellular Carcinoma Patients: A Systematic Review and Meta-Analysis

**DOI:** 10.3390/cancers12010036

**Published:** 2019-12-20

**Authors:** Antonio Facciorusso, Mohamed A. Abd El Aziz, Rodolfo Sacco

**Affiliations:** 1 Department of Medical Sciences, Section of Gastroenterology, University of Foggia, 71122 Foggia, Italy; antonio.facciorusso@virgilio.it; 2 Department of Surgery, Faculty of Medicine, University of Arizona, Tucson, AZ 85719, USA; mabdelmaksoud@email.arizona.edu or; 3 Department of Surgery, Mayo Clinic, Rochester, MN 55905, USA

**Keywords:** sorafenib, advanced, hepatocarcinoma, liver cancer, cirrhosis

## Abstract

Regorafenib showed promising results as a second-line agent after sorafenib failure in hepatocellular carcinoma patients. The aim of this meta-analysis was to evaluate the efficacy and safety of regorafenib in hepatocarcinoma patients. A computerized bibliographic search was performed on the main databases. The primary outcome was overall survival. Secondary outcomes were progression-free survival, tumor response, and the adverse events rate. Outcomes were pooled through a random-effects model and summary estimates were expressed in terms of median and 95% confidence interval or rates, as appropriate. One randomized-controlled trial and seven non-randomized studies with 809 patients were included. The great majority of recruited patients were in Child-Pugh A and ECOG 0 stage. Median overall survival was 11.08 months (9.46–12.71) and sensitivity analyses confirmed this finding, with a median survival ranging from 10.2 to 13.8 months. Duration of regorafenib therapy was 3.58 months, whereas median progression-free survival was 3.24 months (2.68–3.86). The pooled objective response rate was 10.1% (7.8–12.5%) while the disease control rate was 65.5% (61.3–69.7%) with no evidence of heterogeneity (I^2^ = 0%; Diarrhea, fatigue, and hand-foot skin reaction were the most frequent adverse events. The current meta-analysis shows that regorafenib represents a valuable and relatively safe therapeutic option in intermediate/advanced hepatocellular carcinomapatients who progress on sorafenib.

## 1. Introduction

Hepatocellular carcinoma (HCC) represents the most common type of malignancy and the leading cause of death in cirrhotic patients [1].

Despite the latest advancements in diagnosis and screening programs in cirrhotics, a great number of patients are still diagnosed in an advanced stage, thus being unsuitable to curative treatments, such as surgery, orthotopic liver transplantation (OLT), or radiofrequency ablation (RFA) [2,3].

For these subjects with unresectable HCC who cannot benefit from loco-regional treatments, the oral multikinase inhibitor sorafenib (Nexavar^®^, Bayer, Leverkusen, Germany) represents the first-line systemic treatment [2,3,4]. However, since the approval of sorafenib in 2008, the lack of effective second-line agents able to improve treatment outcomes after disease progression during sorafenib therapy has represented one of the major pitfalls in the treatment of HCC.

The oral multikinase inhibitor regorafenib (Stivarga^®^, Bayer, Leverkusen, Germany) prevents the activation of several kinases involved in angiogenesis, oncogenesis, metastatic spread, and tumor immunity [5,6] and it is approved in the therapy of metastasis from colorectal cancer and advanced gastrointestinal stromal tumors [7,8].

A recent phase III multicenter randomized placebo-controlled trial (RCT) showed evidence of the superiority of regorafenib over placebo in HCC patients that had progressed on sorafenib [9]. Since then, several real-life series were published with promising results on the use of regorafenib after sorafenib failure; hence, a pressing need to systematically assess the efficacy of regorafenib in this setting exists, particularly based on available real-life experiences.

As the prolonged survival observed in other cancers might mainly be related to the development of novel effective therapeutic agents able to target the molecular pathways involved in tumor growth and metastasis [10,11], regorafenib is likely to impact significantly the post-progression survival [12,13] of HCC patients.

In an attempt to address this important point, we performed the current meta-analysis of all available studies testing regorafenib as a second-line agent after sorafenib failure in HCC patients. The primary endpoint was overall survival (OS). Additional endpoints were progression-free survival (PFS), tumor response, and adverse events rate.

## 2. Results

### 2.1. Literature Search

**Figure 1:**
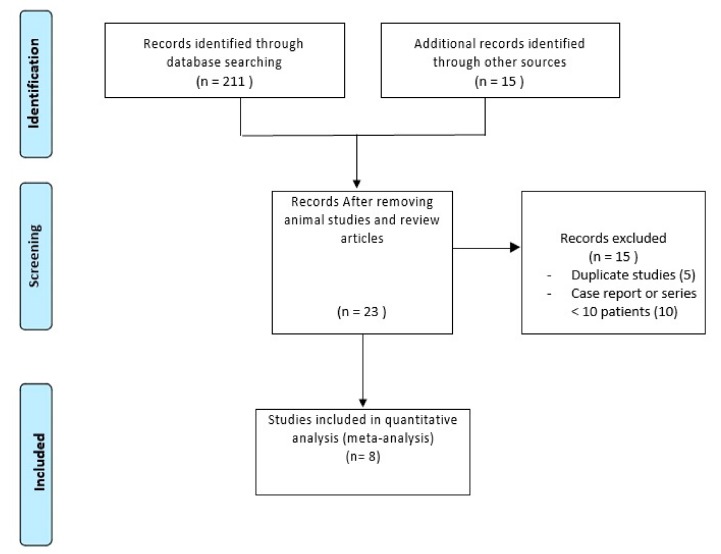
PRISMA flow-chart.

Figure 1 shows the flow chart of the search strategy conducted in this meta-analysis.

Initially, we screened 226 potentially suitable studies. After a preliminary review, 203 studies were excluded, because they were animal studies, comment letters, or descriptive reviews. Then, we excluded 15 potentially appropriate articles, including case reports (<10 patients) or duplicate series. We also excluded an Italian series conducted in the post-OLT setting [14].

Finally, 8 studies were included in the meta-analysis, enrolling 809 patients [9,15,16,17,18,19,20,21].

### 2.2. Characteristics of Included Studies

The main characteristics of included studies are reported in Table 1.

The enrollment period ranged from 2009 to 2019. One study was an RCT [9] and seven non-randomized studies [15,16,17,18,19,20,21]. Two studies were comparative series [9,20], and included studies were conducted mostly in Asia. Two articles were published only as conference proceedings [20,21].

The great majority of recruited patients were in Child-Pugh A and ECOG 0 stage while Barcelona Clinic Liver Cancer (BCLC) stage C was prevalent across the included studies. Viral hepatitis was the most common etiology of the underlying liver disease (Table 1).

The methodological characteristics and quality of included articles are detailed in Supplementary Table S1. Four studies (one RCT and three non-randomized studies) [9,15,16,18] were rated as high quality while the other reports were assessed mainly as moderate quality.

### 2.3. Overall Survival

Overall survival was reported in seven studies [9,15,16,17,19,20,21].

**Figure 2:**
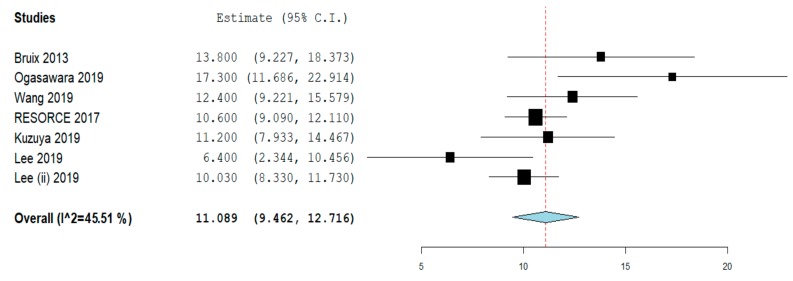
Overall survival.

Median OS was 11.08 months (95% CI: 9.46–12.71), with moderate evidence of heterogeneity (I^2^ = 45.5%; Figure 2). No evidence of publication bias was detected (Supplementary Figure S1A).

Sensitivity analyses conducted according to study quality, patient recruiting (single center vs. multicenter), and study design (RCT vs. non-randomized) confirmed the aforementioned findings, with a median OS ranging from 10.2 to 13.8 months (Table 2).

The duration of regorafenib therapy was reported in four studies [9,15,16,17] and median treatment duration was 3.58 months (2.42–4.74; Supplementary Figure S2).

### 2.4. Progression-Free Survival

**Figure 3:**
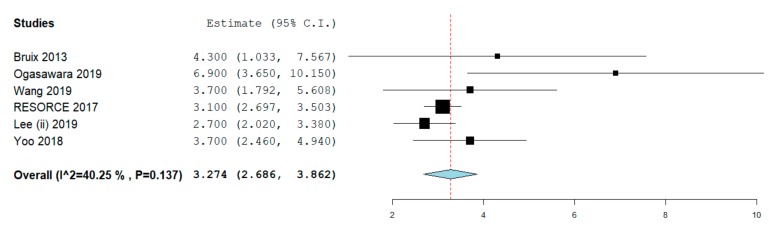
Progression-free survival.

PFS was reported in six studies [9,15,16,17,18,21]. Median PFS was 3.24 months (2.68–3.86), with moderate evidence of heterogeneity (I^2^ = 40.2%; Figure 3). Funnel plot and Begg and Mazumdar’s test (*p* = 0.59) did not show any evidence of publication bias (Supplementary Figure S1B).

Again, sensitivity analyses performed based on the above reported features confirmed the results of the main analysis, with a median PFS ranging from 3.27 to 4.4 months (Table 2).

### 2.5. Complete Response

Tumor response was evaluated in five studies [9,15,17,18,21]. Overall, only two patients in the RESORCE trial [9] experienced a complete response, whereas 48 (9.7%) partial responses were reported out of 493 patients enrolled in four studies [9,15,17,18].

**Figure 4:**
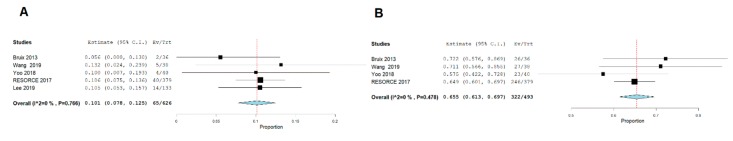
Tumor response. (**A**) Objective response rate; (**B**) Disease control rate.

The pooled analysis of the above reported studies showed an objective response rate as high as 10.1% (7.8–12.5%), with no evidence of heterogeneity (I^2^ = 0%; Figure 4A), while the disease control rate was 65.5% (61.3–69.7%), again with no evidence of heterogeneity (I^2^ = 0%; Figure 4B).

### 2.6. Major Complications

Data on treatment-related complications are reported in the Supplementary Table S2. Diarrhea was the most frequent adverse event, ranging from 27.5% to 55.3% (2.4–10.5% of grade ≥3). Fatigue was experienced by 17.5% to 73.7% of patients while hand-foot skin reaction was reported in more than 50% of treated patients (Supplementary Table S2).

The list of post-regorafenib treatments reported in the included studies is described in the Supplementary Table S3. Among the treatments adopted after regorafenib interruption, lenvatinib was the most frequent third-line systemic agent used.

## 3. Discussion

Hepatocellular carcinoma (HCC) represents the most frequently observed type of cancer and the main cause of tumor-related mortality in cirrhotic patients [1].

Sorafenib, a multikinase inhibitor, has been used since 2008 as a first-line systemic agent in patients with advanced HCC [4]; however, the appropriate treatment in those subjects who are intolerant or progress on sorafenib represents a still unmet need in hepato-oncology.

Regorafenib is an oral multikinase inhibitor of several pro-oncogenic pathways and it showed prolonged survival in patients who experienced tumor progression after the administration of sorafenib in the randomized phase 3 RESORCE trial [9].

However, the real efficacy and safety of regorafenib in real-world practice is still unknown; to the best of our knowledge, our manuscript constitutes the first meta-analysis evaluating the use of regorafenib in HCC patients.

Through a pooled analysis of eight studies, including an RCT and seven non-randomized series, we made several key observations. First, median OS was 11.08 months, a remarkable result considering that regorafenib is used as a second-line agent. This finding represents an encouraging outcome in a setting where effective therapeutic options are lacking, and it is similar to the survival outcomes reported in the SHARP trial with sorafenib as a first-line agent [4]. Of note, the great majority of recruited patients were in Child-Pugh A and ECOG 0 stage, hence the lack of valuable treatment options in patients with decompensated cirrhosis still remains an issue in this field.

Second, median PFS was 3.24 months, which corresponds to the median treatment duration with regorafenib. The lack of effective third-line options in those patients who progress on regorafenib had an impact on the survival outcomes observed in the included studies; this aspect is likely to represent an important research field in the future.

Third, the pooled rates of the objective response and disease control were 10.1% and 65.5%, respectively. Fourth, a number of treatment-related adverse events were registered, including diarrhea, fatigue, and hand-foot skin reaction. Although a wide range of patients experienced such events, less than 10% of treated subjects reported serious complications related to regorafenib treatment. The eventual correlation between adverse event occurrence and treatment response, as already reported with sorafenib [22,23], is unclear, as only the study by Wang et al. performed a regression analysis able to demonstrate the correlation between hand-foot skin reaction and OS [17].

Of note, all of the included studies recruited patients who progressed on sorafenib, therefore the eventual role of regorafenib in the first-line setting is unknown. Further studies are needed in order to evaluate the competitive role of regorafenib with other systemic agents [24], in particular sorafenib, and with transarterial radioembolization in intermediate/advanced HCC patients with portal vein invasion [25,26,27].

Although a specific post-progression survival analysis after regorafenib treatment was not reported in the included studies, lenvatinib was the most frequent third-line systemic agent used after treatment interruption.

Therefore, our results, based mainly on a real-world assessment of regorafenib efficacy, confirm the promising favorable outcomes observed with the preliminary trials, in particular the landmark RESORCE trial [9]. This should enhance the use of regorafenib as a second-line agent in advanced HCC patients.

Unfortunately, specific inflammation markers, such as the systemic immune-inflammation index (SII) or neutrophil-to-lymphocyte ratio (NLR) [28], or adjuvant pharmacological treatments (such as antidiabetic drugs) [29] able to influence regorafenib outcomes have not been studied yet and this aspect should represent a further research field in the future.

There are some limitations to our study. First, the low number of comparative studies did not allow the direct comparison with other available treatments in intermediate/advanced HCC. Second, the majority of included studies were non-randomized series, which may introduce patient selection bias. Third, the relatively low number of treated patients did not enable the efficacy of regorafenib in specific subgroups to be explored, such as Child-Pugh B or BCLC B patients. However, it should be noted that regorafenib was recently introduced in the clinical practice, therefore the current experience is still limited mainly to “optimal” patients in Child-Pugh A and ECOG 0 stage. Further research is needed to address this point properly.

Despite these limitations, our study has a number of strengths. It is the first meta-analysis published in the field and the low/moderate evidence of heterogeneity, as well as the rigorous sensitivity analysis, renders the findings of our study robust and reliable.

## 4. Materials and Methods

### 4.1. Search Strategy and Selection Criteria

A literature search was conducted on PubMed/Medline, Embase, Google Scholar, and Cochrane library databases using the following key words: “Hepatocellular carcinoma”, “liver cancer”, “HCC”, and “regorafenib”. An additional manual search was performed by checking the references of all the main review articles and conference proceedings in the field, in order to retrieve possible additional studies.

Eligible studies were RCTs, prospective or retrospective cohort, and case-control studies reporting on the use of regorafenib in HCC patients until October 2019. The search was restricted to English language articles. Studies were excluded if they were case reports (<10 patients) or animal studies. In the case of duplicate studies or overlapping series, only the last recent publication was included. Included studies were selected independently by two investigators (AF and MAEA). Disagreements were solved by discussion and following a third opinion (RS).

The study quality was rated based on the Cochrane Collaboration’s tool for assessing the risk of bias [30] for RCTs and the Newcastle–Ottawa scale for observational studies [31].

### 4.2. Outcomes

The primary outcome was overall survival (OS), computed from the start of regorafenib therapy. Secondary outcomes were progression-free survival (PFS, computed from the start of regorafenib therapy until the evidence of tumor progression), tumor response, and the adverse events rate.

Complete response was defined as complete necrosis of tumoral nodules assessed at radiological imaging, whereas partial response was defined as at least a 30% decrease in the sum of the longest diameter of target lesions [32]. The objective response rate (ORR) was defined as the sum of the rates of the complete response + partial response while the disease control rate (DCR) was defined as the sum of the rates of the complete response + partial response + stable disease.

### 4.3. Statistical Analysis

Study outcomes were pooled through a random-effects model based on thee DerSimonian and Laird test [33], and results are reported as median and 95% CI for time-to-event data and rates for categorical outcomes.

Chi-square and I² tests were used for the assessment of heterogeneity and *p* < 0.10 for chi-square test and I^2^ < 20% were interpreted as low-level heterogeneity.

Probability of publication bias was assessed using funnel plots and with Begg and Mazumdar’s test.

Safety data were inconsistently reported; hence, they were analyzed descriptively.

Sensitivity analysis was conducted according to the study design (whether RCT or retrospective), quality of included studies (low/moderate versus high), and patient recruitment (single center versus multicenter).

All statistical analyses were conducted using the OpenMeta [Analyst] software (http://www.cebm.brown.edu/openmeta/download.html). For all calculations, a two-tailed *p* value of less than 0.05 was considered statistically significant.

## 5. Conclusions

The current meta-analysis showed that regorafenib represents a valuable and relatively safe therapeutic option in intermediate/advanced HCC patients who progress on sorafenib.

Further RCTs are needed in order to confirm these results.

## Figures and Tables

**Table 1:** Characteristics of the included studies.

Study	Design; Country; Recruitment Period	Intervention	Sample Size (n)	Age (years)	Sex (male)	CP (A/B/C)	ECOG PS (0/≥1)	BCLC Stage (A/B/C)	Etiology of Liver Disease (Viral)	Baseline AFP
**Bruix, 2013 [15]**	Phase II, single arm, open label; multicenter; 2009–2012	160 mg orally once daily for the first 3 weeks of each 4-week cycle, followed by 1 week off treatment for: median (range) weeks: 19.5 (2–103)	36	61 (40–76)	32 (89%)	36 (100%)/0/0	28 (78%)/8 (22%)	0/4 (11%)/32 (89%)	HBV: 14 (39%), HCV: 13 (36%)	
**Ogasawara, 2019 [16]**	Retrospective study: Multicenter, Japan; before March 2018	160 mg regorafenib orally once per day for 3 weeks, followed by 1 week of no treatment for each cycle. 30 patients (68.2%) had initial dose of regorafenib of 160 mg; for 5.7 months (95% CI: 1.82–9.5)	44	71 (60–85)	38 (86.4%)	40 (91%)/4(9%)/0	≤1: 44 (100.0)	C: 34 (77.3)	HBV: 7 (15.9%), HCV: 15 (34.1%)	>400 ng/mL: 17 (38.6)
**Wang 2019 [17]**	Retrospective study; single center, Japan; July 2017 to June 2019	160 mg orally once per day for 3 weeks, followed by 1 week of no treatment for each cycle; for median duration of 2.6 months	38	75 (31–88)	32 (84%)	33 (87%)/5 (13%)/0	17 (45%)/21 (55%)	0/17(45%)/21 (55%)	HBV: 7 (18%), HCV: 16 (43%)	Median (range), ng/mL: 174.2 (2.6–448620); Baseline AFP >400: 16 (42%)
**Yoo 2018** [18]	Retrospective study; multicentre; Korea; April 2017 to August 2017		40	62 (39–83)	36 (90%)	36 (90%)/3 (8%)/1 (2%)	7 (18%)/33 (82%)	0/6 (15%)/34 (85%)	HBV: 27 (67%), HCV: 2 (5%)	≥400 ng/mL: 16 (40%)
**RESORCE trial, 2017 [9]**	Randomized, double-blind, placebo-controlled, phase III trial; multinational (21 countries; 152 centers) study; May 14, 2013 to December 31, 2015		Regorafenib: 379; Placebo: 194	Median (IQR) years: Regorafenib group: 64 (54–71), Placebo group: 62 (55–68)	Regorafenib group: 333 (88%), Placebo group: 171 (88%)	Regorafenib: 373 (98%)/5 (1%)/0; Placebo group: 188 (97%)/6 (3%)/0	Regorafenib: 247 (65%)/132 (35%). Placebo: 130 (67%)/64(33%)/0	Regorafenib: 1 (< 1%)/53 (14%)/325 (86%). Placebo: 0/22 (11%)/172 (89%)	Regorafenib group: *HBV: 143 (38%), *HCV: 78 (21%); Placebo group: *HBV: 73 (38%), *HCV: 41 (21%)	≥400 ng/mL: Regorafenib: 162 (43%), Placebo: 87 (45%).
**Kuzuya, 2019 [19]**	Retrospective study; single center; Japan; between June 2011 and December 2016		36	Age <69 years: 19 patients (52.8%)	32 (88.9%)	A: 27 (75%)/ B &C: 9 (25%)	28 (77.8%)/8(22.2%)	B: 9 (25%)		<400 ng/mL: 27 (75%)
**Lee, 2019 [20]**	Retrospective study (propensity score matching); single center; Korea, 2015–2018		103							
**Lee, 2019 (ii) [21]**	Retrospective study; Multicenter; Korea; 2017–2019		133	60 years	112 (84.2%)	111/ 1/ 1			HBV: 91 (68.4%)	

Data are expressed as median (range) or absolute number (percentage) as appropriate. Conference abstracts Abbreviations list: AFP: Alpha-fetoprotein; BCLC: Barcelona Clinic Liver Cancer; CP: Child-Pough; HBV: Hepatitis B virus; HCV: Hepatitis C virus; PS: Performance status

**Table 2:** Sensitivity analysis of the overall survival and progression-free survival. Sensitivity analysis was performed based on (a) study design (randomized trial versus retrospective study), (b) study quality (low/moderate versus high), and (c) patients’ recruitment (single center versus multicenter). Numbers in parentheses indicate 95% CIs.

Variable	Subgroup	No. of Cohorts	No. of Patients	Summary Estimate (95% CI)	Within-Group Heterogeneity (I^2^)
**Overall Survival**					
Study design	Randomized trial	1	379	13.8 (9.2–18.4)	NA
	Retrospective	6	390	11.4 (9.1–13.6)	61%
Study quality	Low/moderate	4	310	10.2 (8.2–12.2)	46.5%
	High	3	459	13.2 (9.2–17.1)	68.5%
Patients recruitment	Single center	3	177	10.2 (6.9–13.5)	63.5%
	Multicenter	4	592	11.6 (9.5–13.7)	60.8%
**Progression-free Survival**					
Study design	Randomized trial	1	379	4.3 (1.03–7.56)	NA
	Retrospective	5	291	3.65 (2.6–4.7)	52%
Study quality	Low/moderate	3	274	3.4 (1.9–4.9)	48.3%
	High	4	499	3.74 (2.6–4.8)	52%
Patients recruitment	Single center	2	158	4.4 (2.08–6.8)	28.2%
	Multicenter	5	632	3.27 (2.6–3.9)	50%

Abbreviation: CI, Confidence Interval.

## Supplementary Materials

The following are available online at https://www.mdpi.com/2072-6694/12/1/36/s1, Figure S1: Pooled analysis of treatment duration. Table S1: Risk of bias assessment and quality of included studies, Table S2: Adverse events reported in the included studies.
