# Supplementary material for: Efficacy of Regorafenib in Hepatocellular Carcinoma Patients: A Systematic Review and Meta-Analysis
Source: Cancers (Basel). 2019 Dec 20;12(1):36. doi: 10.3390/cancers12010036 (PMC7017079; doi:10.3390/cancers12010036)

Supplementary Table 1. Risk of bias assessment and quality of included studies.

<table>
<tr><th colspan="9">Observational studies[a]</th></tr>
<tr><th></th><th colspan="2">Selection</th><th colspan="2">Comparability</th><th colspan="2">Outcome</th><th colspan="2">Overall quality</th></tr>
<tr><td>Bruix 2013</td><td colspan="2">***</td><td colspan="2">NA</td><td colspan="2">**</td><td colspan="2">H</td></tr>
<tr><td>Ogasawara 2019</td><td colspan="2">***</td><td colspan="2">NA</td><td colspan="2">**</td><td colspan="2">H</td></tr>
<tr><td>Wang 2019</td><td colspan="2">**</td><td colspan="2">NA</td><td colspan="2">**</td><td colspan="2">M</td></tr>
<tr><td>Yoo 2018</td><td colspan="2">***</td><td colspan="2">NA</td><td colspan="2">**</td><td colspan="2">H</td></tr>
<tr><td>Kuzuya 2019</td><td colspan="2">**</td><td colspan="2">NA</td><td colspan="2">**</td><td colspan="2">M</td></tr>
<tr><td>Lee 2019</td><td colspan="2">*</td><td colspan="2">**</td><td colspan="2">*</td><td colspan="2">L</td></tr>
<tr><td>Lee 2019 (ii)</td><td colspan="2">*</td><td colspan="2">NA</td><td colspan="2">*</td><td colspan="2">L</td></tr>
<tr><th colspan="9">Randomized controlled trial [b]</th></tr>
<tr><th></th><th>1</th><th>2</th><th>3</th><th>4</th><th>5</th><th>6</th><th>7</th><th></th></tr>
<tr><td>RESORCE 2017</td><td>L</td><td>L</td><td>L</td><td>L</td><td>L</td><td>L</td><td>L</td><td>H</td></tr>
</table>

L, low; H, high; U, unclear; M, moderate; NA, Not Applicable.

[a] Study quality assessment performed by means of Newcastle/Ottawa scale (each asterisk represents if the respective criterion within the subsection was satisfied)

[b] Cochrane Collaboration's tool for assessing the risk of bias across 7 domains: 1 (Random sequence generation), 2 (Allocation concealment), 3 (Blinding of participants and personnel), 4 (Blinding of outcome assessment), 5 (Incomplete outcome data), 6 (Selective reporting) and 7 (Other bias).

Supplementary Table 2. Adverse events reported in the included studies

	Bruix 2013 (total=36)	RESORCE trial Regorafenib (Total =374)	Placebo (Total = 163)	Ogasawara, 2019 (total = 44)	Wang 2019 (Total = 38)	Yoo 2018 (total = 40)
Any Adverse event						
Any Grade	35 (97.2%)	346 (92.5%)	100 (61.3%)			
≥3	21 (58.3%)	187 (50%)	32 (19.6%)			
Diarrhea						
Any Grade	19 (52.8%)	125 (33.4%)	18 (11.04%)	23 (52.3%)	21 (55.3%)	11 (27.5%)
≥3	2 (5.6%)	9 (2.4%)	0	3 (6.8%)	4 (10.5%)	0
Fatigue						
Any Grade	19 (52.8%)	110 (29.4%)	37 (22.7%)	21 (47.7%)	28 (73.7%)	7 (17.5%)
≥3	6 (16.7%)	24 (6.4%)	3 (1.8%)	2 (4.5%)	1 (2.6%)	0
Hand–foot skin reaction						
Any Grade	19 (52.8%)	196 (52.4%)	13 (7.98%)	30 (68.2%)	25 (65.8%)	27 (67.5%)
≥3	5 (13.9%)	47 (12.6%)	1 (0.6%)	30 (68.2%)	2 (5.3%)	3 (7.5%)
Hypothyroidism						
Any Grade	15 (41.7%)					
≥3	0					
Anorexia						
Any Grade	13 (36.1%)	88 (23.5%)	12 (7.36%)	20 (45.4%)	24 (63.16%)	6 (15%)
≥3	0	10 (2.7%)	0	1 (2.3%)	3 (7.89%)	0
Hypertension						
Any Grade	13 (36.1%)	87 (23.26%)	9 (5.5%)	19 (43.18%)	18 (47.4%)	5 (12.5%)
≥3	1	49 (13.1%)	6 (3.6%)	2 (4.5%)	3 (7.9%)	3 (7.5%)
Nausea						
Any Grade	12 (33.3%)	40 (10.7%)	13 (7.98%)			
≥3	0	1 (0.27%)	0			
Voice changes						
Any Grade	10 (27.8%)	34 (3.1%)	0	12 (27.3%)		
≥3	0	0	0	0		
Constipation						
Any Grade	9 (25%)					
≥3	0					
Headache						
Any Grade	7 (19.4%)					
≥3	0					
Weight loss						

Any Grade	7 (19.4%)	27 (7.2%)	3 (1.8%)		16 (42.1%)	
≥3	0	4 (1.07%)	0		0	
Proteinuria						
Any Grade	6 (16.7%)					
≥3	1 (2.8%)					
Oral mucositis						
Any Grade	5 (13.9%)	42 (11.2%)	5 (3.1%)			
≥3	1 (2.8%)	4 (1.07%)	1 (0.6%)			
Vomiting						
Any Grade	5 (13.9%)	27 (7.22%)	5 (3.1%)			
≥3	0	1 (0.27%)	0			
Abdominal pain						
Any Grade	4 (11.1%)	34 (9.1%)	5 (3.1%)			
≥3	1 (2.7%)	5 (1.3%)	0			
Anemia						
Any Grade	4 (11.1%)	23 (6.14%)	2 (1.2%)	11(25%)		
≥3	1 (2.7%)	18 (4.8%)	6 (3.7%)	0		
Fever						
Any Grade	4 (11.1%)	14 (3.7%)	4 (2.5%)			
≥3	0	0	0			
Hyperbilirubinemia						
Any Grade	4 (11.1%)	70 (18.7%)	7 (4.3%)	7 (16%)	11(28.9%)	1 (2.5%)
≥3	2 (5.6%)	25 (6.7%)	4 (2.5%)	1 (2.3%)	0	1(2.5%)
Hyperthyroidism						
Any Grade	4 (11.1%)					
≥3	1 (2.7%)					
Mood Alteration, depression						
Any Grade	4 (11.1%)					
≥3	0					
Hypophosphatemia						
Any Grade	2 (5.6%)	22 (5.9%)	2 (1.2%)			
≥3	2 (5.6%)	18 (4.8%)	1 (0.6%)			
Elevated AST level						
Any Grade		48 (12.8%)	15 (9.2%)	20 (45.5%)	20 (52.6%)	2 (5%)
≥3		19 (5.1%)	10 (6.1%)	6 (13.6%)	2 (5.26%)	2 (5%)
Hypoalbuminemia						
Any Grade		9 (2.4%)	0	14 (31.8%)		
≥3		2 (0.5%)	0	2 (4.5%)		
Erythema Multiform						
Any Grade				13 (29.5%)		
≥3				2 (4.5%)		
Elevated serum Amylase						
Any Grade				7 (16%)		
≥3				1 (2.3%)		
Thrombocytopenia						
Any Grade		19 (5.1%)	2 (1.2%)	7 (16%)		4 (10%)
≥3		8 (2.1%)	0	2 (4.5%)		1 (2.5%)
Elevated ALT level						
Any Grade		29 (7.8%)	8 (4.9%)		19 (50%)	1 (2.5%)

≥3		12 3.2%)	8 (4.9%)		2 (5.26%)	1 (2.5%)
Neutropenia						
Any Grade						1 (2.5%)
≥3						1 (2.5%)
Constipation						
Any Grade		24 (6.4%)	3 (1.8%)			
≥3		0	0			
Ascites						
Any Grade		8 (2.1%)	1 (0.6%)			
≥3		3 (0.8%)	1(0.6%)			
Limb edema						
Any Grade		12 (3.2%)	1 (0.6%)			
≥3		1 (0.27%)	0			
General disorders and administration site conditions, other						
Any Grade		8 (2.1%)	2 (1.2%)			
≥3		5 (1.3%)	1 (0.6%)			
Investigations, other						
Any Grade		18 (4.8%)	0			
≥3		1 (0.27%)	0			
Back Pain						
Any Grade		2 (0.5%)	2 (1.2%)			
≥3		1 (0.27%)	0			
Cough						
Any Grade		4 (1.7%)	2 (1.2%)			
≥3		0	0			

Supplementary Table 3. Post-regorafenib treatments reported in the included studies

**Study**	**Post-regorafenib treatment**
Bruix 2013	Not reported
Ogasawara 2019	17 patients lenvatinib; 3 patients transarterial chemoembolization (TACE); 2 patients hepatic arterial chemotherapy; 1 patient radiation therapy
Wang 2019	10 patients lenvatinib; 4 patients oral cytotoxic therapies; 1 patient cabozantinib; 1 patient ramucirumab; 5 patients transarterial therapies; 1 patient radiation therapy
Yoo 2018	5 patients nivolumab; 1 patient doxorubicin; 2 patients sperimental therapy
RESORCE trial 2017	Not reported
Kuzuya 2019	Not reported
Lee 2019 (i)	Not reported
Lee 2019 (ii)	Not reported

Supplementary Figure 1. Funnel plots for detecting the risk of publication bias in A) overall survival analysis; B) progression-free survival analysis

A

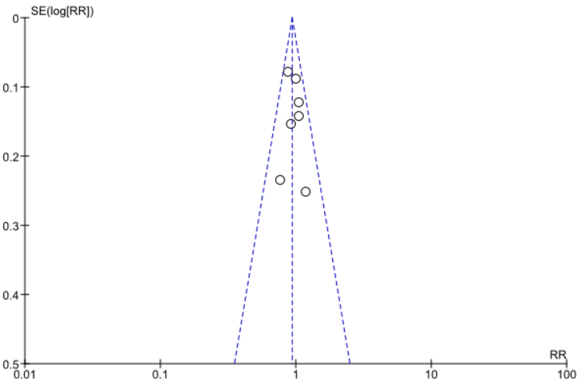

B

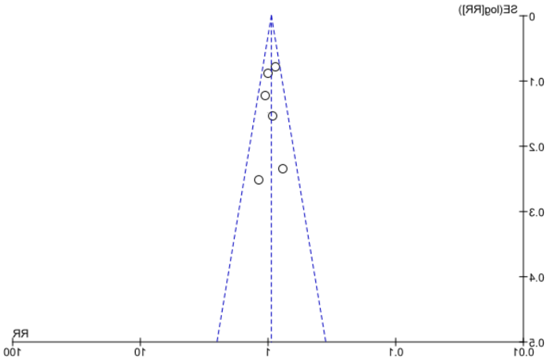

Supplementary Figure 2. Pooled analysis of treatment duration.

Studies	Estimate (95% C.I.)
Bruix 2013	4.900 (1.633, 8.167)
Ogasawara 2019	5.700 (1.859, 9.541)
Wang 2019	2.600 (0.692, 4.508)
RESORCE 2017	3.600 (1.788, 5.412)
**Overall (I^2=0 % , P=0.422)**	**3.586 (2.424, 4.748)**

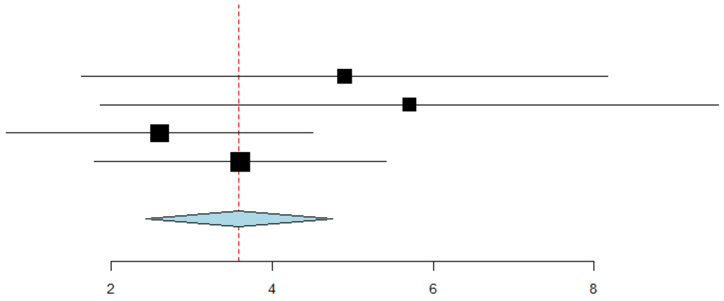

Supplement: Supplementary file 1 [file cancers-12-00036-s001.pdf]
